# Testing the effects of presentation modality and presence of similarity percentages in the automatic image recognition system in Italy

**DOI:** 10.3389/fpsyg.2026.1794679

**Published:** 2026-04-07

**Authors:** Aicia Naser, Giovanni Tessitore, Roberto Atzeni, Fabrizio Corrado Adamo, Alessandro Santirocchi, Giacomo Rogliero, Vincenzo Cestari, Clelia Rossi-Arnaud

**Affiliations:** 1 Deparment of Psychology, Sapienza University of Rome, Rome, Italy; 2 PhD Programme in Behavioural Neuroscience, Sapienza University of Rome, Rome, Italy; 3 Central Directorate of the Forensic Police and Cyber Security, Rome, Italy

**Keywords:** automated face recognition system, law enforcements, presentation modality, recognition accuracy, similarity percentages

## Abstract

Automatic Face Recognition Systems (AFRS) are commonly used by police operators to compare the image of an unknown subject (target face) with those already recorded in specific databases. In operational forensic workflows, AFRS support identification by generating a lineup through a one-to-many (1: N) search, comparing a probe image against a large biometric gallery in which candidates are ranked by similarity scores. The purpose of the present study was to determine how different interface configurations, specifically image presentation modality and the inclusion of similarity percentages, impact human decision-making and identification accuracy. No study has investigated the impact of different layouts on the accuracy of detecting the correct potential candidate. Here, we tested the effects of presentation modality and the presence/absence of similarity percentages on the recognition performance of untrained participants in an AFRS. Participants studied a target face for 5 s and were then asked to recognize it from a list of 50 faces, which may or may not have included the target. The faces were presented in one of three formats: simultaneously (8 images per slide), semi-sequentially (4 images per slide), or sequentially (1 image per slide) and participants were always given the opportunity to compare the selected image with the target face (direct comparison). Each image was either shown with or without a system-generated similarity percentage. When similarity percentages were shown, participants were instructed either to use or ignore them. Results showed that, for target-absent lists, false alarms were fewer with the simultaneous layout than with the semi-sequential or sequential ones. Direct comparisons were also significantly lower with the simultaneous condition, and, in this condition, participants who made more direct comparisons were more prone to false alarms in identifying targets in target-present lists. The research identifies which model best mitigates cognitive biases and improve decision-making accuracy. The results provide a new evidence-based standard for AFRS interface design, offering specific recommendations for law enforcement.

## Introduction

Face matching and face recognition play a fundamental role in identity verification, Law Enforcement investigations, and border security. Despite the common perception that facial recognition is a natural and highly accurate ability in humans, research has shown that performance can vary significantly between different individuals and operational contexts (e.g., White et al., 2015; Towler et al., 2019). While often used as interchangeable terms, face matching and face recognition refer to slightly different abilities, served by distinct processes. Specifically, face recognition involves identifying a known individual from memory, whereas face matching refers to the direct comparison of two facial images (or the comparison of a target image to several potential candidates: Bobak et al., 2016), without prior memory encoding (Fysh and Bindemann, 2018).

Studies by Bindemann et al. (2012) highlighted that the ability to compare unknown faces is subject to considerable errors, with critical implications for biometric and forensic recognition. White et al. (2015), for example, found that participants made over 50% errors when matching unfamiliar adult faces, and over 60% when matching images of children. Similarly, Bobak et al. (2016) analyzed individual differences in face matching and showed that some individuals, known as super-recognizers, possess exceptional skills in facial recognition.

Two crucial factors that affect face matching accuracy are the degree of experience and the impact of contextual variables. Regarding the first factor, the studies by White et al. (2014) have shown that targeted training can improve performance in face matching and face recognition tasks, although the cognitive limitations of human operators remain a risk factor. For the second factor, it is well known that conditions such as lighting, face angle, and image quality can significantly affect face matching performance (White et al., 2017). These factors are particularly relevant in real-world scenarios, such as airport security checks and surveillance systems, where suboptimal lighting, varying facial orientations, and low-resolution images can compromise identification accuracy.

Consequently, the reliability of automatic recognition technologies has been extensively analyzed, revealing their potential and limitations (White et al., 2015). This has prompted the scientific community to develop new algorithms and improve strategies to minimize errors in facial identification processes.

Currently, several automatic facial recognition systems are employed by Law Enforcements and public safety contexts. Cognitec, for example, specializes in facial recognition solutions, frequently implemented in airports and critical infrastructures. The Face++ system, developed by the Chinese company Megvii, has likewise gained prominence due to its deep-learning capabilities, while NEC NeoFace is widely adopted across multiple countries for identity verification and access control. Turning to Italy, the only system used by Law Enforcement is the SARI (*Sistema Automatico di Riconoscimento Immagini*) Enterprise, which enables Law Enforcement agencies to support the identification process of potential candidates through extensive facial image databases. Different presentation layout can be used to show the candidate list. For example in a simultaneous grid layout for 1: N matching task a ‘probe’ image is displayed alongside a ranked gallery of candidates, typically accompanied by numerical similarity (Schroff et al., 2015). Despite high algorithmic precision (Grother et al., 2019), this layout may encourage relative judgments where users pick the ‘best’ match from a set rather than absolute judgments (Wells, 1984), potentially increasing false-positive rates. Furthermore, because non-specialized practitioners often perform similarly to untrained novices (White et al., 2014), the influence of visual cues and cognitive load (Sweller, 1988) on decision-making is a critical concern. Other presentation modalities that can be used to visualize the output list comprise a quasi-sequential modality (where the images are presented 4 at a time, one below the other).

Specifically, the SARI system allows operators to compare a probe image to those contained in the A. F. I. S. (Automated Fingerprint Identification System) database – which includes more than 10 million images. In no cases the result of SARI is a match but the output is always a list of 50 images of potential candidates, obtained through an algorithm that specifies the order of presentation (from the most similar to the least similar) and provides, for each image, a similarity percentage.

The system thus functions as a face-matching support tool and is designed to assist the police operator in searching and filtering potential suspects within the AFIS mugshot database, identifying those whose facial features most closely resemble those in the probe image. It supports the operator’s decision-making process but does not make any decisions autonomously.

When a suspect’s image is manually selected—typically from CCTV footage—it is then uploaded into the system—faithfully reproduced in our experiment—which generates a list of potential candidates. The operator can select the presentation layout for the images, choosing between viewing 4 images at a time (quasi-sequential) or 8 images at a time (simultaneous). The operator navigates the candidate list using a mouse and can perform a direct comparison between the suspect and any candidate by clicking on the selected image. This action triggers a comparison screen that displays both images side-by-side. By using the mouse, the operator can return to the main gallery and repeat this process as many times as deemed necessary until a final match or non-match judgment is reached and only after having analyzed all the candidates.

Since no previous study has tested the accuracy of the face matching performance of human operators using AFRS systems, the present experiment was specifically aimed at exploiting the above methodology to examine the effects of presentation modality and the presence/absence of similarity percentages on the identification accuracy of untrained student participants. The use of non-expert participants was aimed at isolating general decision-making mechanisms affected by interface layout and similarity metrics and not at evaluating specialized professional judgment, also considering that most operators are not experts in face-matching. Focusing on a non-expert sample allowed the identification of intrinsic cognitive vulnerabilities that arise independently of domain-specific expertise. The resulting findings provide an empirical basis for the development of standardized design guidelines and operational protocols aimed at reducing bias and improving decision accuracy across different levels of operator expertise.

Studies on the difference between the sequential and simultaneous presentations have been almost always concerned with face recognition and have typically used lineups with a variable number of images during the test phase (from 6 to 10; Finley et al., 2015). With simultaneous presentation, witnesses can see all the candidates and, consequently, can be oriented to think that at least one of the faces must correspond to the culprit (Flowe and Ebbesen, 2007). Based on these relative judgments, it is possible that subjects select as the culprit the image that is closest to their memory trace and that, if that subject is removed from the list, they indicate as the culprit the second closest image (Wells et al., 1998; Wells et al., 1993). The seminal study by Lindsay and Wells (1985) has indeed demonstrated that sequential and simultaneous presentations lead to similar proportions of correct identification in target-present lists; however, false alarms in target-absent lists were substantially lower with the sequential than with the simultaneous presentation. Later studies have generally confirmed these findings, although a meta-analysis by Steblay et al. (2011) concluded that both hit rates (i.e., correct identifications) and false alarms were lower in the sequential than in the simultaneous lineup procedure, suggesting that the sequential presentation may simply induce participants to use a more conservative response criterion (Finley et al., 2015). Importantly for present purposes, it is unclear whether this pattern can be extended to face-matching tasks. We are unaware of previous studies involving a direct comparison between simultaneous and sequential face-matching procedures and indirect evidence is rather mixed. Megreya and Burton (2006, Exp.1), for example, reported that the overall accuracy in a simultaneous task in which participants had to match a target face to an array of 10 potential candidates was 82%; specifically, correct responses in target-present arrays (i.e., hits) were about 88%, whereas false alarms in target-absent arrays were about 23%. When the task was modified to involve a match between a target face and a single image (sequential version; Exp.4), the corresponding percentages were 79% (overall accuracy), 81% (hits), and 22% (false alarms). These results would suggest a slight reduction in correct identifications in the sequential version (as happens in classical face recognition tasks: Steblay et al., 2011) and no difference in false alarms. However, a later study from the same authors offered a different picture (Megreya and Burton, 2006). In this case, the overall accuracy in the simultaneous 1-to-10 matching task was about 66% (Exp.2), with 71% of correct identifications in target-present arrays and 37% of false alarms in target-absent arrays. The corresponding percentages in the sequential 1-to-1 matching task were about 84%, with 85% of correct identifications in target-present arrays and 15% of false alarms in target-absent arrays. This data would therefore suggest a strong increase in correct identifications in the sequential presentation, together with a substantial reduction in false positives (paralleling the results obtained in face recognition tasks: Steblay et al., 2011).

Despite the existence of studies comparing human and machine performance in face-matching tasks (Carragher and Hancock, 2023), there is currently a lack of empirical evidence concerning the role of similarity percentages. To date, virtually no data in the literature can be used as a basis for predicting their effects. Although similarity percentages are a common feature of automated face-matching systems, no previous study has systematically examined their impact on performance in face-matching tasks. This point is particularly important in the context of AFRS systems, because police officers are sometimes instructed to disregard the similarity percentages provided by the system. One aim of the present study was therefore to determine whether participants were actually able to ignore these percentages. To this purpose, we included three different conditions: a baseline condition in which the similarity percentages were not provided, a standard condition in which the percentages were provided, and participants were told that they represented a system-generated measure of the similarity of each image with the target face, and an ‘ignore’ condition, in which participants were explained the meaning of the percentages but were told to disregard them. The reason to include the latter condition is that individuals often struggle to ignore automatically processed information, even when explicitly instructed to do so. This phenomenon has been extensively documented in cognitive psychology, particularly in studies on attentional control and inhibition. The classical Stroop effect, for example, demonstrates the difficulty of suppressing the automatic elaboration of the semantic meaning of words, even when the task requires to respond solely to the color in which they are printed (MacLeod, 1991). This difficulty can be further understood through the lens of Dual Process Theory (Kahneman, 2003). According to this framework, the presentation of salient information, such as similarity percentages, likely triggers ‘System 1’, an intuitive, fast, and relatively effortless processing mode. Because System 1 operates automatically and outside of conscious control, the similarity data may be integrated into the decision-making process before the analytical ‘System 2’ can intervene to evaluate or inhibit it. Relying on System 1 significantly reduces the cognitive and memory load associated with the task, but often at the expense of accuracy and a more critical evaluation of the information.

In the directed forgetting paradigm participants are instructed to remember some words, while simultaneously forgetting other words. In a later recall task, the typical finding is that to-be-ignored words are remembered significantly worse than to-be-remembered words (Costanzi et al., 2021). However, the inhibition effect is far from being completely effective (i.e., participants do remember many words that were instructed to forget), is subject to release (Basden et al., 1993), and does not occur in all tasks (i.e., it is often absent or strongly reduced in implicit tasks: McKinney and Woodward, 2004). Based on these findings, we hypothesize that similarity percentages, when presented, may affect identification decisions, even when operators are explicitly asked to ignore them. Clarifying the extent of this influence is critical, as it may reveal unintended cognitive biases in forensic applications and inform guidelines to enhance the reliability of face-matching decisions.

To this end, the present study addressed two primary research questions. First, we aimed to determine which presentation layout facilitates higher identification accuracy in a 1: N face-matching task. Second, we investigated whether the presence of similarity percentages significantly influences identification judgments, even when participants are explicitly instructed to disregard them. Based on the aforementioned frameworks, we hypothesized that simultaneous layouts would lead to higher accuracy but that similarity percentages would exert a biasing effect on decision-making regardless of the instructions provided.

## Methods

### Participants

A total of 180, including students and nonstudents, took part in the present study.

“The inclusion of a non-expert sample facilitated the identification of inherent strengths and critical vulnerabilities in both the system’s design and its matching procedure.”

Participants were randomly assigned to nine experimental groups (*n* = 20 per group). Each group completed the task independently under one of the experimental conditions. Overall, the sample included 120 females and 60 males (age: M = 23.8 years, SD = 3.1). Random assignment ensured a balanced distribution of demographic characteristics across the groups. Sample size was determined based on prior power analysis conducted using G*Power (Faul et al., 2007), assuming a medium effect size (*f* = 0.25), with power set at 0.80 and *α* = 0 0.05. This required a minimum of 20 participants per group, consistent with recommendations for detecting medium-sized effects in within- and between-subjects comparisons. All subjects aged between 18 and 35 are included in the sample. The exclusion criteria were presence of pathologies that substantially compromise visual capacity. Request for normal or corrected vision (glasses or lenses).

### Materials

The experimental materials consisted of 10 photographic line-ups, each composed of 50 frontal-view images of Caucasian adult faces, including both male and female individuals (1/3 male and 2/3 female).

For methodological purposes, we included only Caucasian adult faces to control for intervening factors such as own-race bias. Five line-ups included the target face (target-present condition), while the remaining five did not (target-absent condition). Each line-up was presented under three different presentation modes: true sequential, semi-sequential, and simultaneous. In total, 500 unique facial images were used, with 50 different faces assigned to each line-up. Each presentation mode was combined with one of three information conditions regarding numerical cues: presence of percentages, presence of percentages accompanied by an explicit recommendation to disregard them, and absence of percentages. The facial stimuli used in the study were selected from two publicly accessible databases: the Generated Photos repository,^1^ which provides AI-generated synthetic faces, and the UT Dallas Face Database,^2^ containing high-quality images of real faces. All target faces were real, with the exception of the face used in line-up number six, which was taken from the UT Dallas dataset. To ensure a robust evaluation of the AFRS layout while adhering to legal and ethical constraints regarding the use of biometric databases, we employed a controlled AI-generation process to supplement the Dallas Face Database. To maintain high ecological validity, each AI-generated face was derived from a real source image, allowing the system to produce variations that retain the biometric proportions and textures of actual human faces while ensuring a diverse and statistically sufficient sample size for the recognition system. Once the set of target and filler images was defined, the 10 line-ups were constructed using an AFRS using ADA Face algorithm (Kim et al., 2022). The software was used to automatically generate balanced arrays by combining the selected stimuli based on their visual similarity to the target face, according to predefined experimental conditions. For each target, the software returned a ranked list of faces ordered by descending similarity percentages, enabling the construction of line-ups with controlled levels of visual resemblance between the target and the fillers. All images were standardized format and presented against a neutral white background to ensure uniformity in lighting, contrast, and visual composition. The experiment was administered using PowerPoint, on Lenovo laptops with 15.6-inch screens and a resolution of 1920 × 1080 pixels. Participants completed the task individually in a quiet room, maintaining a standardized viewing distance of approximately 55 cm. The experimental interface was navigated by participants via mouse and keyboard input. To date, no formal validation procedure has been conducted on the specific selection of images used in the present study. However, all stimuli were chosen to ensure basic comparability in terms of demographic features (e.g., age, gender, ethnicity) and were standardized in visual appearance. Each image had a resolution of 640 × 480 pixels. The study was conducted in accordance with the ethical standards of the Declaration of Helsinki and was approved by the Ethics Committee for Transdisciplinary Research (CERT) of the Sapienza University of Rome. Ethical approval was granted under protocol number 165/2024. All participants provided informed consent prior to participation and were informed of their right to withdraw from the study at any time. Participation was voluntary and anonymous, and no personally identifiable information was collected.

### Procedure

At the beginning of each trial, participants were shown a target face that they were instructed to memorize. Immediately afterward, they were presented with a line-up list and asked to identify the previously seen target face, if present. This procedure was repeated across 10 trials, with each participant completing 10 different line-ups randomly assigned to the experimental conditions. Half of the line-ups included the target face (target-present), while the other half did not (target-absent), allowing for the assessment of both correct identifications of a potential match and false alarms.

**Figure 1:**
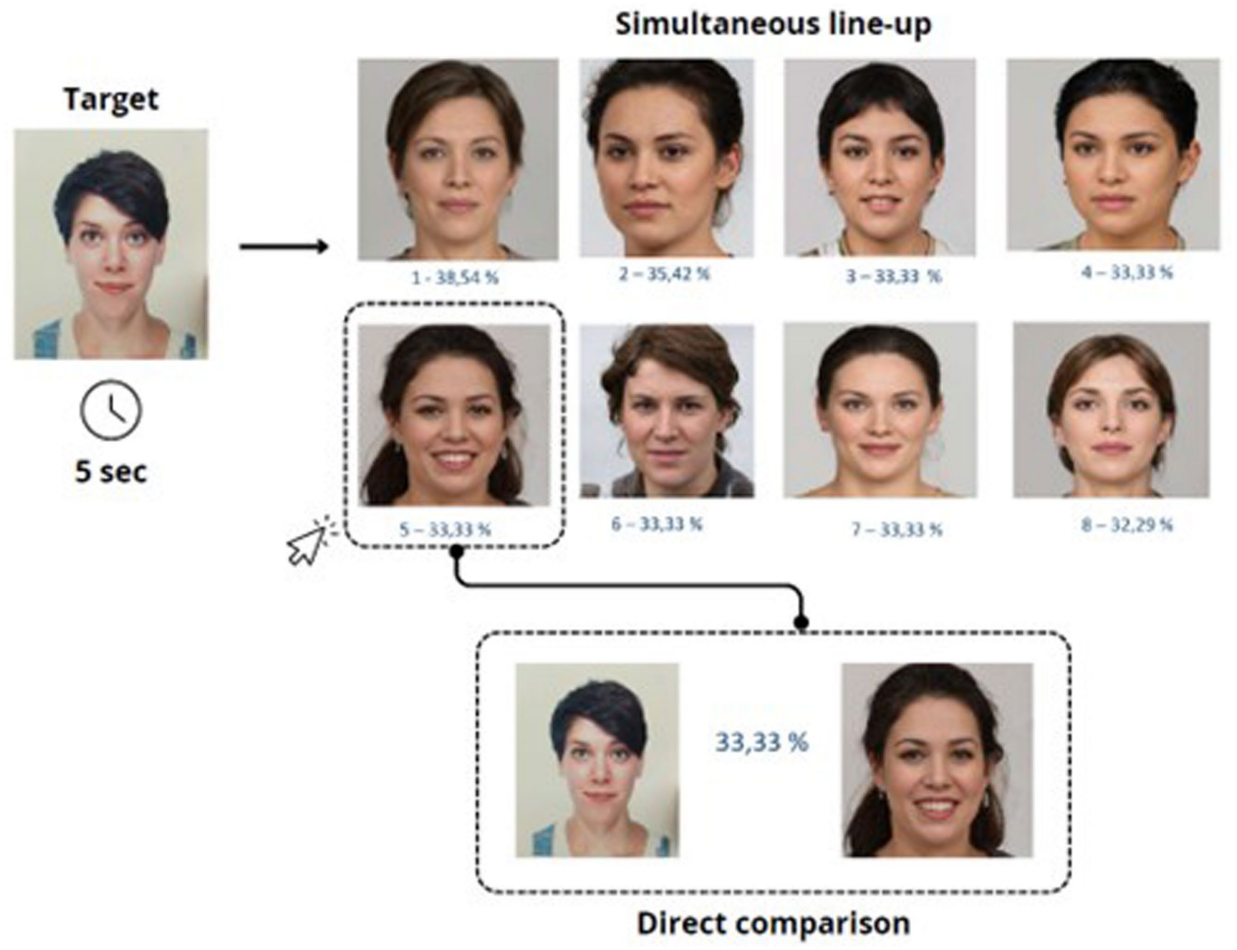
All six faces were presented at the same time on a single screen, arranged in two horizontal rows of three images each. Participants could view and compare the faces freely.

**Figure 2:**
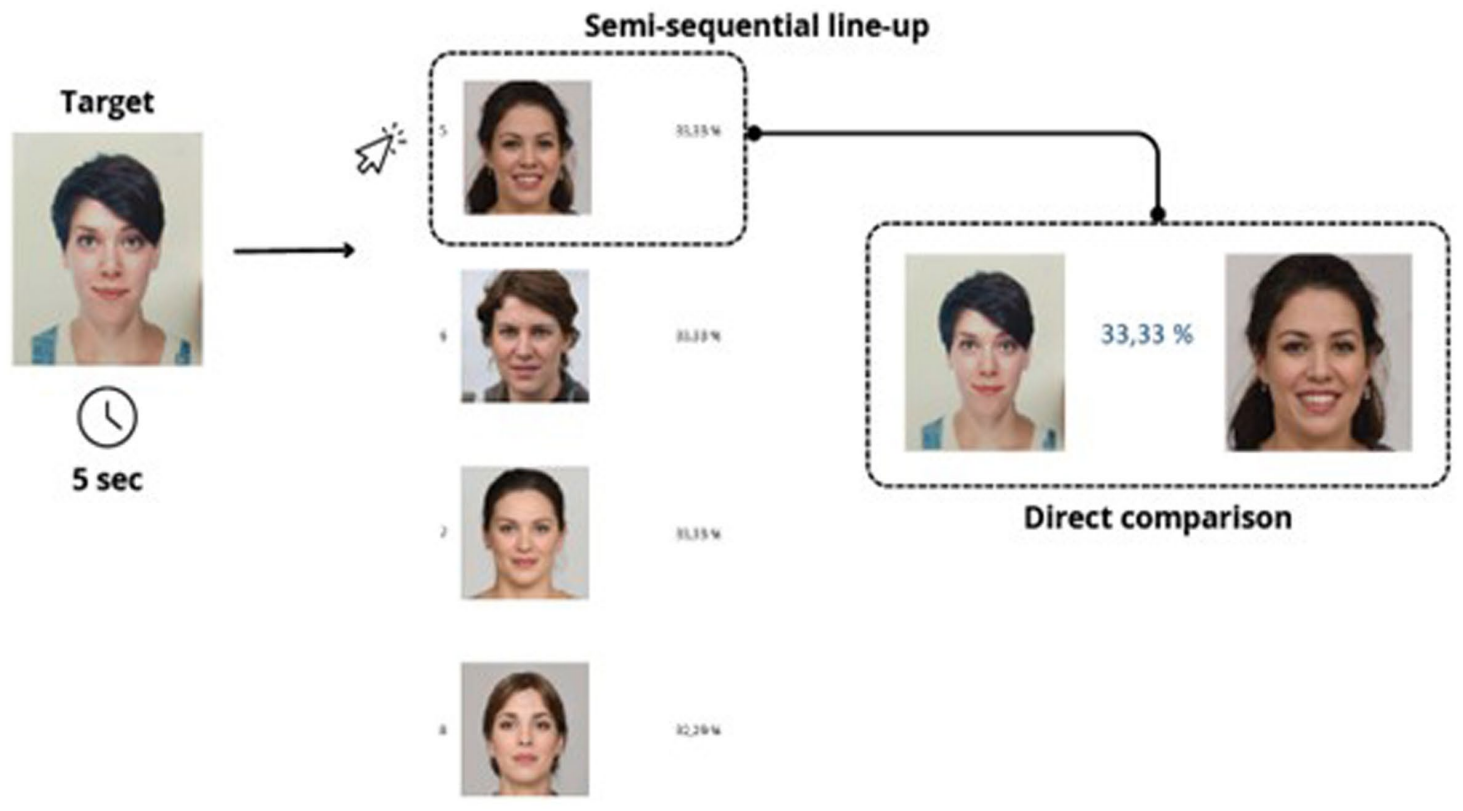
Four faces were displayed vertically on a single screen. Participants could scroll freely back and forth to view all the items.

**Figure 3:**
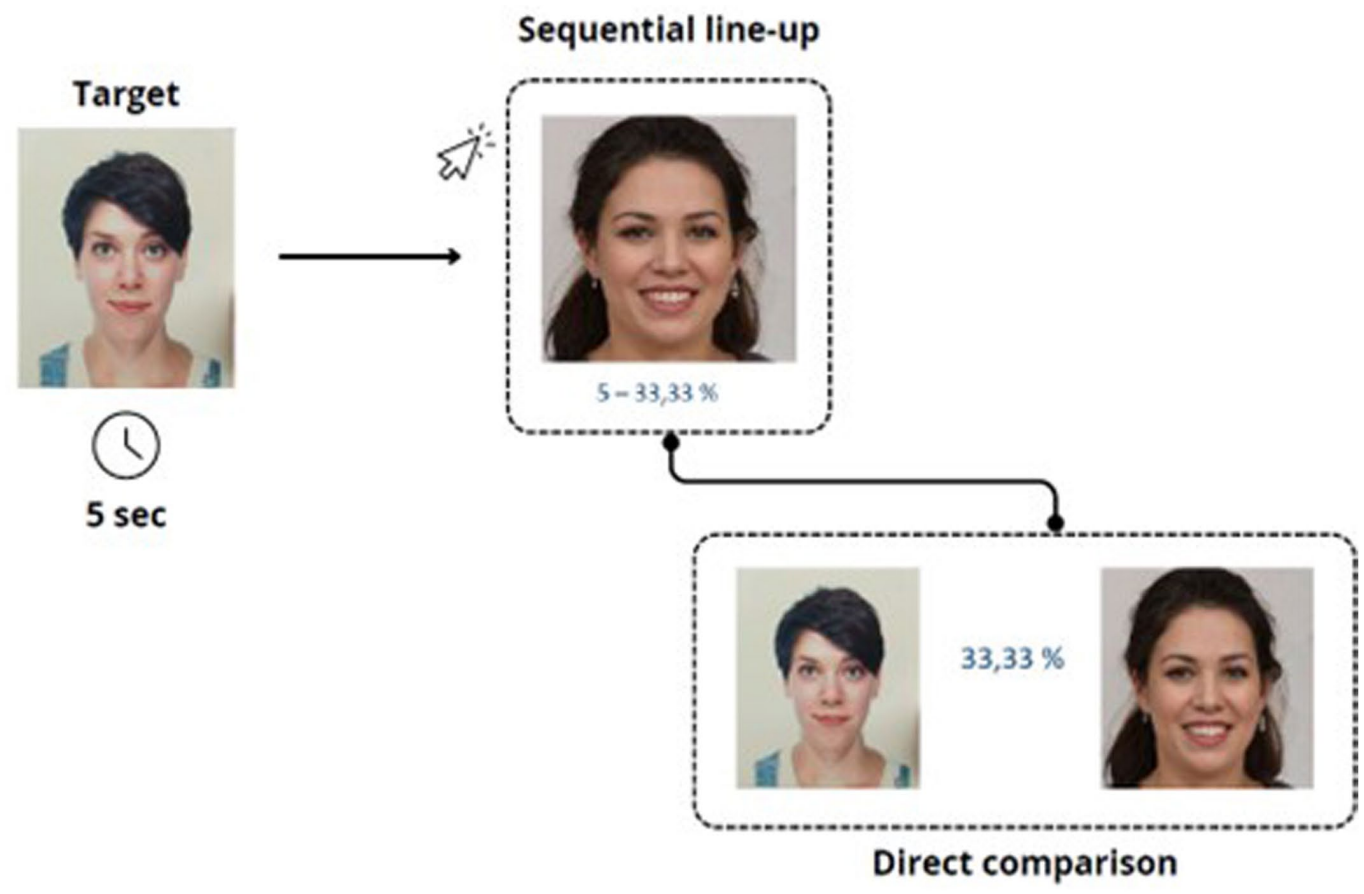
Each face was shown individually in a fixed order, one per screen, with participants viewing them one at a time.

In the simultaneous condition, all six faces were displayed at once on a single screen, arranged in two horizontal rows of three images each (see Figure 1). Participants could examine the entire set freely. In the semi-sequential condition, four faces were shown vertically, one below the other, on a single screen (see Figure 2). Participants could scroll freely back and forth within the list. In the true sequential condition, each of the faces was presented individually, one per screen, in a fixed order (see Figure 3).

Participants could move forward or backward through the sequence using navigation buttons.

In all three presentation modes, participants had the option to activate a direct comparison between the currently selected face and the original target image on a new screen. Upon clicking on a face, a new window displayed the two images side by side to facilitate the recognition decision. Participants completed the task individually on a computer and were not given feedback on their responses. Although the task was presented as self-paced, a maximum duration of 3 min and 30 s per trial was enforced to standardize data collection and avoid excessively long response times. Participants were not informed of the exact time limit, but the experimenter manually stopped the trial if it was exceeded. The maximum duration of the experiment was therefore approximately 33 min.

### Data analysis

To examine the impact of presentation layouts and similarity information on face-matching performance, we conducted a series of Mixed-Design Analyses of Covariance (ANCOVA) and Analyses of Variance (ANOVA).

Specifically, the primary experimental design was a 3 (Type of Presentation: simultaneous, semi-sequential, sequential) × 3 (Type of Condition: no percentages, percentages without instructions, percentages with instructions) factorial model. In the overall analysis of correct identifications, participants’ age was included as a covariate to control for its potential influence on facial processing abilities.

Dependent variables included identification accuracy (proportions of correct identifications and filler identifications, analyzed separately for target-present and target-absent lists) and direct comparisons performed between the target image and candidates.

Post-hoc comparisons were conducted using the Bonferroni adjustment to maintain the family-wise error rate. Additionally, simple effects analyses were employed to decompose significant two-way interactions. Finally, Pearson’s correlation coefficients were calculated to investigate the relationship between the frequency of direct comparisons and identification performance across all experimental conditions.

## Results

### Simultaneous layout outperforms other layouts in correct identifications

**Figure 4:**
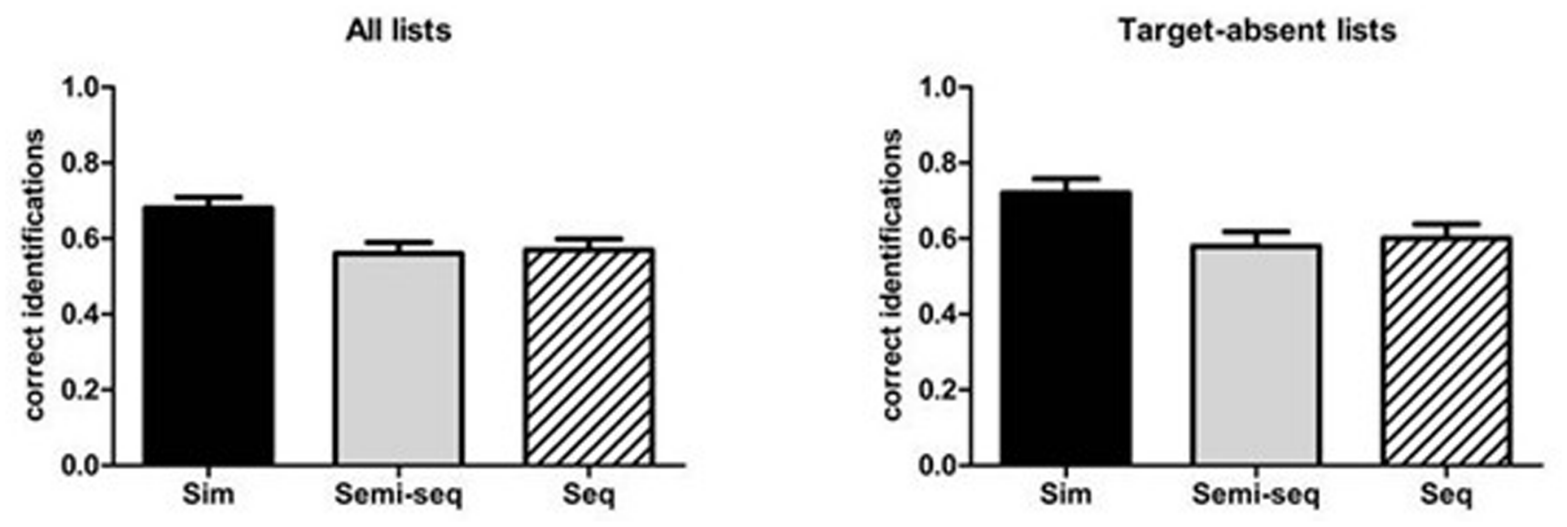
Mean proportions of correct identifications for all lists (left panel) and target-absent lists (right panel), as a function of type of presentation. The bars indicate standard errors.

The total proportions of correct identifications (see Table 1), considering both target-present and target-absent lists, were submitted to a 3 (Type of presentation: simultaneous, semi-sequential, sequential) × 3 (Type of condition: no percentages, percentages without instructions, percentages with instructions) ANCOVA, in which both factors were manipulated between subjects and participants’ age was included as a covariate. The analysis revealed a significant main effect of Type of presentation [*F*(2, 170) = 4.24, MSE = 0.051, *p* = 0.016, *η*_p_^2^ = 0.048]. The post-hoc comparisons, with the Bonferroni adjustment, indicated that the proportions of correct identifications were higher for the simultaneous (*M* = 0.67) than for the other two presentations (*M* = 0.57, *p* = 0.044 for the sequential presentation and *M* = 0.56, *p* = 0.032 for the semi-sequential presentation; see Figure 4, left panel). The main effect of Type of condition [*F*(2, 170) = 1.52, MSE = 0.051, *p* = 0.22, *η*_p_^2^ = 0.018] and the two-way interaction [*F*(4, 170) = 1.70, MSE = 0.051, *p* = 0.15, *η*_p_^2^ = 0.039] did not reach the significance level.

**Table 1:** Mean proportions of correct identifications, as a function of type of presentation and type of condition.

	No percentages	Percentages no instructions	Percentages with instructions
All lists			
Simultaneous	0.65 (0.22)	0.65 (0.17)	0.73 (0.16)
Semi-sequential	0.63 (0.26)	0.51 (0.24)	0.55 (0.28)
Sequential	0.65 (0.17)	0.58 (0.25)	0.48 (0.21)
Target-present lists			
Simultaneous	0.62 (0.23)	0.60 (0.25)	0.68 (0.25)
Semi-sequential	0.66 (0.29)	0.43 (0.24)	0.57 (0.32)
Sequential	0.56 (0.25)	0.59 (0.29)	0.49 (0.29)
Target-absent lists			
Simultaneous	0.69 (0.30)	0.70 (0.27)	0.78 (0.18)
Semi-sequential	0.61 (0.34)	0.59 (0.33)	0.54 (0.35)
Sequential	0.76 (0.17)	0.57 (0.33)	0.48 (0.27)

Standard deviations are reported in parenthesis.

### No differences in correct identifications for target-present lists

Limiting the analyses to the proportions of correct identifications for target-present lists, the same ANOVA as above revealed no main effects [Type of presentation: *F*(2, 170) = 1.89, MSE = 0.075, *p* = 0.15, *η*_p_^2^ = 0.022; Type of condition: *F*(2, 170) = 1.11, MSE = 0.075, *p* = 0.33, *η*_p_^2^ = 0.013] and no interaction between the two factors [*F*(4, 170) = 1.82, MSE = 0.075, *p* = 0.12, *η*_p_^2^ = 0.041].

### Simultaneous layout improves correct responses in target-absent lists compared to semi-sequential

For target-absent lists, the ANOVA on the proportions of correct responses found a significant main effect of Type of presentation [*F*(2, 170) = 3.70, MSE = 0.086, *p* = 0.027, *η*_p_^2^ = 0.042]. The post-hoc comparisons indicated that the proportions of correct identifications were higher for the simultaneous (*M* = 0.72) than the semi-sequential presentation (*M* = 0.58, *p* = 0.040) – all other comparisons were non-significant (*p* > 0.090; see Figure 4, right panel).

### Simultaneous layout slightly reduces filler identifications

**Figure 5:**
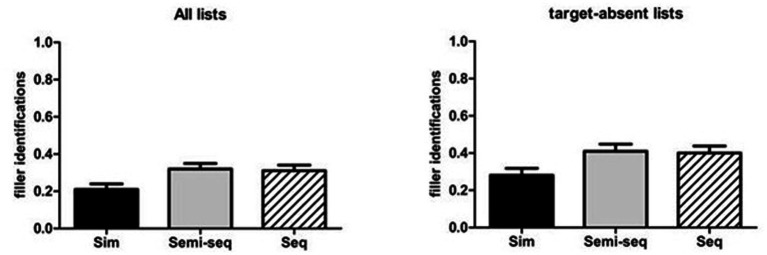
Mean proportions of filler identifications for all lists (left panel) and target-absent lists (right panel), as a function of type of presentation. The bars indicate standard errors.

The ANOVA on the total proportions of filler identifications (see Table 2) revealed a significant main effect of Type of presentation [*F*(2, 170) = 3.65, MSE = 0.054, *p* = 0.028, *η*_p_^2^ = 0.041]. The post-hoc comparisons indicated that the proportions of filler identifications were marginally lower with the simultaneous presentation (*M* = 0.21) than with the other two presentations (*M* = 0.31, *p* = 0.059 for the sequential presentation and *M* = 0.32, *p* = 0.062 for the semi-sequential presentation; see Figure 5, left panel). The main effect of Type of condition [*F*(2, 170) = 1.11, MSE = 0.054, *p* = 0.33, *η*_p_^2^ = 0.013] and the two-way interaction [*F*(4, 170) = 1.80, MSE = 0.054, *p* = 0.130, *η*_p_^2^ = 0.041] were not significant.

**Table 2:** Mean proportions of filler identifications, as a function of type of presentation and type of condition.

	No percentages	Percentages no instructions	Percentages with instructions
All lists			
Simultaneous	0.26 (0.20)	0.20 (0.18)	0.18 (0.15)
Semi-sequential	0.27 (0.25)	0.32 (0.24)	0.36 (0.29)
Sequential	0.21 (0.12)	0.33 (0.26)	0.40 (0.25)
Target-present lists			
Simultaneous	0.22 (0.17)	0.10 (0.13)	0.14 (0.22)
Semi-sequential	0.16 (0.22)	0.23 (0.20)	0.27 (0.31)
Sequential	0.19 (0.23)	0.22 (0.30)	0.29 (0.29)
Target-absent lists			
Simultaneous	0.31 (0.28)	0.30 (0.27)	0.22 (0.18)
Semi-sequential	0.39 (0.34)	0.41 (0.33)	0.46 (0.35)
Sequential	0.24 (0.17)	0.44 (0.32)	0.52 (0.27)

Standard deviations are reported in parenthesis.

### No differences in filler identifications for target-present lists across layouts or conditions

The ANOVA on filler identifications for target-present lists (see Table 2) found no main effects [Type of presentation: *F*(2, 170) = 1.85, MSE = 0.059, *p* = 0.16, *η*_p_^2^ = 0.021; Type of condition: *F*(2, 170) = 0.75, MSE = 0.059, *p* = 0.47, *η*_p_^2^ = 0.009] and no interaction [*F*(4, 170) = 1.22, MSE = 0.059, *p* = 0.30, *η*_p_^2^ = 0.028].

### Simultaneous layout reduces false identifications in target-absent lists

The ANOVA on false alarms for target-absent lists (see Table 2) revealed a significant main effect for Type of presentation [*F*(2, 170) = 3.78, MSE = 0.085, *p* = 0.025, *η*_p_^2^ = 0.043]. The post-hoc analyses, with the Bonferroni correction, indicated that false identifications with the Simultaneous presentation (*M* = 0.28) were significantly lower than those with the Semi-sequential presentation (*M* = 0.41, *p* = 0.040), and marginally lower than those with sequential presentation (*M* = 0.40, *p* = 0.076; see Figure 5, right panel). The main effect of Type of condition and the two-way interaction did not reach the significance level [*F*(2, 170) = 1.32, MSE = 0.085, *p* = 0.26, *η*_p_^2^ = 0.015 and *F*(4, 170) = 2.12, MSE = 0.085, *p* = 0.080, *η*_p_^2^ = 0.048, respectively].

### Sequential layout increases total direct comparisons, especially with percentages plus instructions

**Figure 6:**
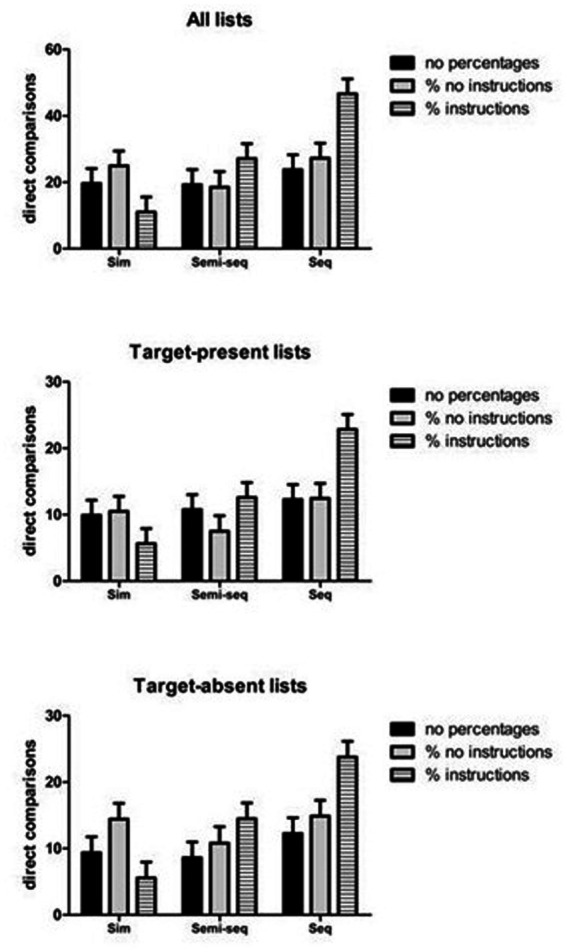
Interaction between type of presentation and type of condition on the number of direct comparisons. The bars indicate standard errors.

The ANOVA on the total number of direct comparisons revealed a significant main effect of Type of presentation [*F*(2, 170) = 8.04, MSE = 404.49, *p* < 0.001, *η*_p_^2^ = 0.086]. The post-hoc comparisons indicated that the total number of direct comparisons were higher with the sequential (*M* = 32.56) than the simultaneous (*M* = 18.54, *p* = 0.001) and semi-simultaneous presentations (*M* = 21.65, *p* = 0.011; the latter two conditions did not differ between them: *p* = 1.00). In addition, the two-way interaction was also significant [*F*(4, 170) = 4.47, MSE = 404.49, *p* = 0.002, *η*_p_^2^ = 0.095]. The follow-up analyses of simple effects (see Figure 6, upper panel) revealed that the effect of Type of presentation was significant in the condition with percentages and instructions [*F*(2, 170) = 15.46, MSE = 404.49, *p* < 0.001, *η*_p_^2^ = 0.154], but not in the other two conditions [*F*(2, 170) = 0.30, MSE = 404.49, *p* = 0.73, *η*_p_^2^ = 0.004 and *F*(2, 170) = 0.95, MSE = 404.49, *p* = 0.38, *η*_p_^2^ = 0.011, respectively]. In the condition with percentages and instructions, the post-hoc comparisons confirmed that participants made more direct comparisons with the sequential presentation (*M* = 46.63) than with the simultaneous (*M* = 11.04, *p* < 0.001) and semi-sequential (*M* = 27.12, *p* = 0.008) presentations; furthermore, participants made more comparisons with the semi-sequential than with the simultaneous presentation (*p* = 0.040). The same analysis indicated that the main effect of Type of Condition was significant with the sequential presentation [*F*(2, 170) = 7.45, MSE = 404.49, *p* = 0.001, *η*_p_^2^ = 0.081], but not with the other two presentation modalities [*F*(2, 170) = 1.11, MSE = 404.49, *p* = 0.33, *η*_p_^2^ = 0.13 and *F*(2, 170) = 2.41, MSE = 404.49, *p* = 0.093, *η*_p_^2^ = 0.028, respectively]. With the sequential presentation, participants made more direct comparisons in the condition with percentage and instructions (*M* = 46.63) than in the condition without percentages (*M* = 27.26, *p* = 0.001) or with percentages and no instructions (*M* = 27.12, *p* = 0.008).

### Sequential layout boosts direct comparisons in target-present lists only under percentages plus instructions

The ANOVA on the number of direct comparisons in target-present lists revealed a significant main effect of Type of presentation [*F*(2, 170) = 8.41, MSE = 101.26, *p* < 0.001, *η*_p_^2^ = 0.090] and a significant two-way interaction [*F*(4, 170) = 3.93, MSE = 101.26, *p* = 0.004, *η*_p_^2^ = 0.085]. The follow-up analyses of simple effects revealed a pattern of results comparable with that obtained in the overall analysis (see Figure 6, middle panel). Specifically, the main effect of Type of presentation was significant in the condition with percentages and instructions [*F*(2, 170) = 14.57, MSE = 101.26, *p* < 0.001, *η*_p_^2^ = 0.146], but not in the other two conditions [*F*(2, 170) = 0.28, MSE = 101.26, *p* = 0.75, *η*_p_^2^ = 0.003 and *F*(2, 170) = 1.16, MSE = 101.26, *p* = 0.31, *η*_p_^2^ = 0.013, respectively]. In the condition with percentages and instructions, the post-hoc comparisons confirmed that participants made more direct comparisons with the sequential presentation (*M* = 22.83) than with the simultaneous (*M* = 5.63, *p* < 0.001) and semi-sequential (*M* = 12.58, *p* = 0.005) presentations. The main effect of Type of condition was not significant [*F*(2, 171) = 1.87, MSE = 102.06, *p* = 0.15, *η*_p_^2^ = 0.021]. Moreover, the main effect of Type of Condition was significant with the sequential presentation [*F*(2, 170) = 7.16, MSE = 101.26, *p* = 0.001, *η*_p_^2^ = 0.078], but not with the other two presentation modalities [*F*(2, 170) = 1.26, MSE = 101.26, *p* = 0.28, *η*_p_^2^ = 0.015 and *F*(2, 170) = 1.38, MSE = 101.26, *p* = 0.25, *η*_p_^2^ = 0.016, respectively]. With the sequential presentation, participants made more direct comparisons in the condition with percentage and instructions (*M* = 22.83) than in the condition without percentages (*M* = 12.29, *p* = 0.003) or with percentages and no instructions (*M* = 12.46, *p* = 0.004).

### Direct comparisons in target-absent lists are enhanced by sequential layouts and reduced by simultaneous layouts

The ANOVA on the number of direct comparisons in target-absent lists showed a significant main effect of Type of presentation [*F*(2, 170) = 7.60, MSE = 113.11, *p* = 0.001, *η*_p_^2^ = 0.082], a marginal main effect of Type of condition [*F*(2, 170) = 2.85, MSE = 113.11, *p* = 0.060, *η*_p_^2^ = 0.033], and a significant two-way interaction [*F*(4, 170) = 4.26, MSE = 113.11, *p* = 0.003, *η*_p_^2^ = 0.091]. In line with the overall analysis, the follow-up analyses of simple effects (see Figure 6, lower panel) revealed that the effect of Type of presentation was significant in the condition with percentages and instructions [*F*(2, 170) = 14.41, MSE = 113.11, *p* < 0.001, *η*_p_^2^ = 0.145], but not in the other two conditions [*F*(2, 170) = 0.66, MSE = 113.11, *p* = 0.51, *η*_p_^2^ = 0.008 and *F*(2, 170) = 0.82, MSE = 113.11, *p* = 0.44, *η*_p_^2^ = 0.010, respectively]. In the condition with percentages and instructions, the post-hoc comparisons confirmed that participants made more direct comparisons with the sequential presentation (*M* = 23.76) than with the simultaneous (*M* = 5.55, *p* < 0.001) and semi-sequential (*M* = 14.48, *p* = 0.019) presentations; furthermore, they made more comparisons with the semi-sequential than with the simultaneous presentation (*p* = 0.029). The same analysis indicated that effect of Type of condition was significant with the sequential [*F*(2, 170) = 6.38, MSE = 113.11, *p* = 0.002, *η*_p_^2^ = 0.070] and simultaneous presentations [*F*(2, 170) = 3.48, MSE = 113.11, *p* = 0.033, *η*_p_^2^ = 0.039], but not with the semi-sequential presentation [*F*(2, 170) = 1.54, MSE = 113.11, *p* = 0.21, *η*_p_^2^ = 0.018, respectively]. With the sequential presentation, participants made more direct comparisons in the condition with percentages and instructions (*M* = 23.76) than in the conditions without percentages (*M* = 12.26, *p* = 0.002) or with percentages and no instructions (*M* = 14.88, *p* = 0.028); however, with the simultaneous presentation, participants made *less* comparisons in the condition with percentages and instructions (*M* = 5.55) than in the condition with percentages and no instructions (*M* = 14.45, *p* = 0.028).

### Higher direct comparison frequency correlates with poorer performance

To determine whether the number of direct comparisons performed affected the proportions of correct and filler identifications, we computed Pearson’s correlations. The results indicated that the total number of direct comparisons were negatively correlated with the total number of correct identifications (*r* = −0.18, *p* = 0.018). However, the same correlations for target-present and target-absent lists did not reach the significance level (*r* = −0.13, *p* = 0.083 and *r* = −0.14, *p* = 0.055, respectively). Regarding filler identifications, there were positive correlations with the number of direct comparisons in the overall analysis (*r* = 0.22, *p* = 0.003), as well as in the separate analyses for target-present (*r* = 0.23, *p* = 0.002) and target-absent lists (*r* = 0.15, *p* = 0.050).

## Discussion

### Operational relevance of interface design in AFRS

As AFRS technologies are increasingly used by Law Enforcement agencies, it is crucial to understand how interface designs influence operators’ decisions, since AFRS support identification process and may result in investigations going in the wrong direction based on false suspicions.

Thus, identifying correct procedures and interface configurations, designed to minimize errors and false alarms in experimental settings, may result in systems that maximize correct face matching.

The present study provides the first empirical evidence on how the modality of image presentation and the presence (or absence) of similarity percentages may influence face matching performance in AFSR-like conditions.

### Effects of presentation modality on face-matching performance

Specifically, regarding our first research question (which layout facilitates better identification accuracy) results indicate that the simultaneous presentation layout significantly facilitates performance compared to other modalities.

The simultaneous presentation, in which the entire grid of images is displayed at the same time, allows the operator to visually compare all the faces presented, reducing dependence on serial comparison and limiting the influence of decision biases. In particular, a significant increase in accuracy was observed, in target-absent lists, suggesting that simultaneous viewing helps to avoid forced selections in ambiguous situations. This evidence is consistent with previous research showing that simultaneous presentation limits the witness’s tendency to choose the “most similar” face, rather than rejecting the entire grid in case of doubt, reducing the so-called relative judgment bias (Flowe and Ebbesen, 2007; Wells and Turtle, 1986).

Other studies have confirmed that in simultaneous lineups, participants adopt a face-to-face comparison strategy that promotes a decision-based process for the comparison of stimuli rather than on the isolated mnemonic recall of the perpetrator’s face (Lindsay and Wells, 1985). The holistic approach favored by simultaneous presentation is also supported by neuroscientific evidence: facial processing activates specific cortical regions (e.g., FFA – Fusiform Face Area), whose activity is enhanced in conditions that require simultaneous visual comparison, thus improving the discriminability between similar faces (Haxby et al., 2000). Meta-analyses on facial recognition in lineups suggest that simultaneous presentation, compared to sequential presentation, results in a comparable or higher rate of correct identifications, with fewer false positives in cases where the perpetrator is not present (Steblay et al., 2001; Gronlund et al., 2012). These data further reinforce the validity of using simultaneous grids in AFRS. These results are not consistent with the data reported in the meta-analysis by Steblay et al. (2001) and with the comparative studies by Finley et al. (2015) and Wells et al. (2015). This conflicting evidence could be explained by the fact that our study involves a face matching task, while the above-mentioned studies consider a face recognition task.

### Influence of similarity percentages and procedural instructions

Regarding our second research question (whether similarity percentages influence face-matching judgments) our findings are relevant for operational models and forensic protocols. A key finding concerns the influence of similarity percentages provided by automated systems in the “ignore” condition, where participants made significantly more direct comparisons, particularly under sequential presentation.

This pattern suggests that similarity percentages trigger System 1 (Kahneman, 2003). When participants are explicitly told to ignore this information, it leads to a failure of inhibitory control. The effort to disregard the data actually increases cognitive load and results in maladaptive strategies, such as excessive direct comparisons, without any gain in accuracy. Correlational analyses indeed indicate that a higher number of direct comparisons was associated with a decrease in correct identifications and an increase in filler identifications.

The present study suggests that similarity percentages in face matching tasks may not influence judgment per se, but only under specific conditions and presentation layout. When similarity percentages were accompanied by an explicit instruction to ignore them and a sequential presentation layout, participants engaged in a higher number of direct comparisons. This pattern suggests that the instruction functioned as a cue for increased attentional control and decision caution, encouraging a more accurate strategy.

This interpretation aligns with evidence from decision-making research showing that explicit warnings or task-relevant instructions can induce more conservative response criteria and promote effortful processing (Evans and Stanovich, 2013). The increased number of direct comparisons observed in the condition “sequential presentation, percentages and instruction to ignore” is consistent with a shift toward more controlled strategy rather than automatic responding. However, this increased level of attention did not translate into better performance. Correlational analyses indicate that a higher number of direct comparisons was associated with a decrease in correct identifications and an increase in false alarms. Results thus indicate that similarity information may negatively influence decision-making when this information is associated with explicit “ignore” instructions and a sequential presentation layout. By prompting heightened vigilance and more careful analysis, the instructions may increase cognitive load and promote the adoption of maladaptive strategies, leading to a higher number of false alarms.

No significant differences emerged between the condition in which similarity percentages were presented without instruction and the condition in which no percentages were presented at all. This finding suggests that the presence of similarity percentages, when left uninterpreted, does not necessarily influence decision-making. This information appears to exert an influence when it is made explicit through instructions, becoming salient and reshaping the decision strategy. These data are consistent with models emphasizing adaptive shifts in attentional allocation and metacognitive monitoring in response to task demands (Ackerman and Thompson, 2017).

The above considerations highlight that procedural instructions and operator awareness can influence facial comparison strategies more strongly than numerical similarity information. Evidence shows that specific training and targeted feedback can significantly improve facial recognition accuracy even in non-experts, suggesting that errors stem more from suboptimal cognitive strategies than from intrinsic inability (White et al., 2015, 2017). Expert operators, including trained forensic analysts and super-recognizers, tend to rely less on external numerical cues, adopting a more stable and less suggestible criterion when assessing facial similarity (Towler et al., 2019). Without adequate training, the inclusion of similarity percentages may introduce bias, potentially distorting visual judgment in specific layouts.

### Time pressure and ecological validity

Time pressure represents another factor that can compromise decision accuracy. In time–pressure situations, where of decision making can compromise accuracy, the combination of advanced technology and targeted training is crucial (Bindemann et al., 2016). Nevertheless, in real-world settings, trained operators performing face-matching tasks using AFRS are typically not subject to strict time constraints, allowing for extended deliberation and repeated verification. However, in experimental contexts, the imposition of a time limit serves important methodological functions. First, it helps standardize cognitive load across participants, minimizing inter-individual differences in time-on-task that could confound performance measures (Bindemann et al., 2010). Second, it reduces the likelihood of response strategies based on exhaustive comparisons or over-analysis, which can introduce noise into the measurement of recognition accuracy (Megreya and Burton, 2006). Importantly, while participants were not explicitly informed of the precise time limit, this approach balances naturalistic decision-making with experimental rigor, allowing for spontaneous behavior within a bounded framework. Nevertheless, operators in real-world settings do not work under strict time constraints such as the ones required in an experimental setting, but rather under the pressure and responsibility associated with the consequences of their decisions. Such conditions may differently shape the dynamics of the decision-making process, potentially reducing the ecological validity of the findings.

### Limitations and future directions

The generalizability of our results may be affected by factors beyond the imposed time limit. First, the sample consisted primarily of non-expert participants, and the stimuli were limited to Caucasian faces, restricting applicability to real operational situations. Classic studies on identification procedures emphasize the need to evaluate more ecological and diverse contexts for full validation (Wells and Seelau, 1995; Wells et al., 1998). Another limitation concerns the use of system-generated similarity percentages: since it is unclear whether participants relied on or ignored this information, its actual influence on decision-making remains uncertain.

Future research should involve more heterogeneous samples, including professional forensic analysts and “super-recognizers,” to examine whether expertise mitigates observed biases in the sequential presentation. Super-recognizers outperform trained police officers in face-matching tasks (Russell et al., 2009), making them valuable in security and forensic contexts. However, these abilities are not universal, and automated tools remain essential to support human decision-making (Fysh and Bindemann, 2018). Future studies should also explore the “other-race effect” across different ethnic groups to ensure the reliability of AFRS globally. Testing the paradigm in real operational contexts with professional operators would provide a more accurate assessment of ecological validity and practical applicability.

### Implications for AFRS design, training, and policy

Overall, our study indicates that interface design and procedural instructions are not neutral elements but active components that shape cognitive strategies. Our quantitative data clearly show that the simultaneous layout is the most effective configuration for reducing false identifications in 1: N tasks. Furthermore, the findings regarding similarity percentages for certain layout (mainly sequential presentation) suggest that an operational model of “instructed disregard” is psychologically untenable, as it paradoxically increases cognitive effort while degrading performance accuracy.

The evidence provided by this research, in line with the proposals of Robertson et al. (2016) and Edmond et al. (2015), supports the need to develop more rigorous guidelines on the use of facial identification systems, which should include the standardization of face presentation methods, but also control over the use and interpretation of numerical metrics provided by recognition software if combined with specific presentation layout. Furthermore, operator training should include specific modules on the cognitive processes of facial recognition, the management of biases, and the critical interpretation of similarity percentages generated by automatic systems.

The integration of technological tools with expert human judgment is essential for improving the accuracy of facial identification. Bacci et al. (2021) show that morphological analysis remains a critical component in validating face comparisons and underline the value of an integrated approach that considers both technological and human aspects. The adoption of procedures based on simultaneous presentation could help reduce identification errors, especially in situations where the target subject is not present in the lineup, as also suggested by the comparative studies of Wells et al. (2015) and Finley et al. (2015). However, the inclusion of system-generated information such as similarity percentages must be carefully managed to avoid influence on the operator’s judgment. To this aim, targeted training is crucial (Moreton, 2021).

The study also highlights the importance of designing interface layouts and decision environments that align with the cognitive mechanisms underlying facial matching. Rather than treating the presentation format and supplementary information as neutral elements, these should be seen as active components that shape the way in which practitioners process and assess facial stimuli. In this sense, our results contribute to the development of cognitively informed tools and protocols that improve the reliability of human decisions within semi-automated identification systems.

Research in cognitive ergonomics and human factors highlights the importance of aligning system interfaces with the perceptual and cognitive constraints of human operators (Wickens et al., 2004). In high-stakes tasks such as face matching, the layout of visual elements, the mechanisms for image comparison, and the format of supplementary information (e.g., similarity scores) should be carefully structured to support optimal processing strategies and minimize the likelihood of decision errors (Norman, 1990; Vicente, 2003). For example, presenting multiple candidate faces simultaneously may facilitate holistic comparison and reduce unnecessary cognitive load, whereas overwhelming or ambiguous cues, such as poorly contextualized similarity percentages, can, in non expert-participants and specific layouts, introduce confusion or cognitive bias, especially under time pressure or uncertainty. Our findings support this view, suggesting that even small differences in presentation modality or information saliency can have measurable impacts on presentation modality or information saliency can have measurable impacts on performance. This reinforces the need for AFRS interfaces to be designed and tested with human cognitive architecture in mind, integrating empirical data from experimental psychology to inform evidence-based system improvements.

## Conclusion

In conclusion, despite the limitations outlined above, our findings indicate that interface design choices and procedural instructions critically shape human performance in face-matching tasks, with implications for the deployment of AFRS in operational settings.

A simultaneous grid layout significantly reduces false identifications in 1: N searches, particularly by increasing correct rejections, underscoring the need to align system design with human perceptual and decision-making processes. Conversely, similarity scores, in non-expert participants and specific layouts, might undermine accuracy. These findings call for evidence-based policy and design guidelines to ensure that AFRS interfaces support human judgment in forensic and security contexts.

More broadly, these results contribute to the ongoing debate (see Carragher and Hancock, 2023) on the integration of algorithmic support and human decision-making in security contexts.

## Data Availability

The raw data supporting the conclusions of this article will be made available by the authors, without undue reservation.

## Footnotes

1
https://generated.photos/

2
https://utdallas.box.com/v/facedatabase

## References

[ref1] Ackerman R. Thompson V. A. (2017). Meta-reasoning: monitoring and control of thinking and reasoning. Trends Cogn. Sci. 21, 607–617. doi: 10.1016/j.tics.2017.05.004, 28625355

[ref2] Bacci N. Davimes J. G. Steyn M. Briers N. (2021). Forensic facial comparison: current status, limitations, and future directions. Biology 10:1269. doi: 10.3390/biology10121269, 34943183 PMC8698381

[ref3] Basden B. Basden D. Gargano G. (1993). Directed forgetting in implicit and explicit memory tests: a comparison of methods. J. Exp. Psychol. Learn. Mem. Cogn. 19, 603–616.

[ref4] Bindemann M. Avetisyan M. Blackwell K. (2010). Finding needles in haystacks: identity mismatch frequency and facial identity verification. J. Exp. Psychol. Appl. 16, 378–386. doi: 10.1037/a0021893, 21198254

[ref5] Bindemann M. Avetisyan M. Rakow T. (2012). Who can recognize unfamiliar faces? Individual differences and observer consistency in person identification. J. Exp. Psychol. Appl. 18, 277–291. doi: 10.1037/a0029635 22905851

[ref6] Bindemann M. Fysh M. Cross K. Watts R. (2016). Matching faces against the clock. i-Perception. 7. doi: 10.1177/2041669516672219 PMC5051631 27757219

[ref7] Bobak A. K. Hancock P. J. B. Bate S. (2016). Super-recognisers in action: evidence from face-matching and face memory tasks. Appl. Cogn. Psychol. 30, 81–91. doi: 10.1002/acp.3170, 30122803 PMC6084338

[ref8] Carragher D. J. Hancock P. J. B. (2023). Simulated automated facial recognition systems as decision-aids in forensic face matching tasks. J. Exp. Psychol. Gen. 152, 1286–1304. doi: 10.1037/xge0001310, 36455036

[ref9] Costanzi M. Cianfanelli B. Santirocchi A. Lasaponara S. Spataro P. Rossi-Arnaud C. . (2021). Forgetting unwanted memories: active forgetting and implications for the development of psychological disorders. J. Personalized Med. 11:241. doi: 10.3390/jpm11040241, 33810436 PMC8066077

[ref10] Edmond G. Tangen J. M. Searston R. A. Dror I. E. (2015). Contextual bias and cross-contamination in the forensic sciences: the corrosive implications for investigations, plea bargains, trials and appeals. Law Probab. Risk 14, 1–25. doi: 10.1093/lpr/mgu018

[ref11] Evans J. S. Stanovich K. E. (2013). Dual-process theories of higher cognition: advancing the debate. Perspect. Psychol. Sci. 8, 223–241. doi: 10.1177/1745691612460685 26172965

[ref12] Faul F. Erdfelder E. Lang A. G. Buchner A. (2007). G*power 3: a flexible statistical power analysis program for the social, behavioral, and biomedical sciences. Behav. Res. Methods 39, 175–191. doi: 10.3758/BF03193146 17695343

[ref13] Finley J. R. Roediger H. L. III Hughes A. D. Wahlheim C. N. Jacoby L. L. (2015). *The American journal of psychology*, Vol 128(2), sum 2015 special issue: Alice Healy festschrift. Am. J. Psychol. 128, 173–195. doi: 10.3899/jrheum.090579 26255438

[ref14] Flowe H. D. Ebbesen E. B. (2007). The effect of lineup member similarity on recognition accuracy in simultaneous and sequential lineups. Law Hum. Behav. 31:33. doi: 10.1007/s10979-006-9045-9, 17123159

[ref15] Fysh M. C. Bindemann M. (2018). Human-computer interaction in face matching. Cogn. Sci. 42, 1714–1732. doi: 10.1111/cogs.12633, 29954047 PMC6099365

[ref16] Gronlund S. D. Carlson C. A. Neuschatz J. S. Goodsell C. A. Wetmore S. A. Wooten A. . (2012). Showups versus lineups: an evaluation using ROC analysis. J. Appl. Res. Mem. Cogn. 1, 221–228. doi: 10.1016/j.jarmac.2012.09.003

[ref17] Grother P. Ngan M. Hanaoka K. (2019). Face Recognition Vendor Test (FRVT) Part 2: Identification, NIST Interagency/Internal Report (NISTIR), National Institute of Standards and Technology, Gaithersburg, MD, [online]. doi: 10.6028/NIST.IR.8271

[ref18] Haxby J. V. Hoffman E. A. Gobbini M. I. (2000). The distributed human neural system for face perception. Trends Cogn. Sci. 4, 223–233. 10827445 10.1016/s1364-6613(00)01482-0

[ref19] Kahneman D. (2003). Maps of bounded rationality: psychology for behavioral economics. Am. Econ. Rev. 93, 1449–1475. doi: 10.1257/000282803322655392

[ref20] Kim M. Jain A. K. Liu X. (2022). Adaface: Quality adaptive margin for face recognition. In Proceedings of the IEEE/CVF conference on computer vision and pattern recognition. 18750–18759.

[ref21] Lindsay R. C. Wells G. L. (1985). Improving eyewitness identifications from lineups: simultaneous versus sequential lineup presentation. J. Appl. Psychol. 70:556.

[ref22] MacLeod C. M. (1991). Half a century of research on the Stroop effect: an integrative review. Psychol. Bull. 109, 163–203. 2034749 10.1037/0033-2909.109.2.163

[ref23] McKinney L. C. Woodward A. E. (2004). Remembering what one intended to forget: the lack of directed forgetting effects in implicit memory. Am. J. Psychol. 117, 169–190. doi: 10.2307/4149021, 15209368

[ref24] Megreya A. M. Burton A. M. (2006). Unfamiliar faces are not faces: evidence from a matching task. Mem. Cogn. 34, 865–876. doi: 10.3758/bf03193433, 17063917

[ref26] Moreton R. (2021). Forensic face matching: Procedures and application. Forensic face matching: Research and practice. ed. Bindemann M. Oxford Academic online. doi: 10.1093/oso/9780198837749.003.0007

[ref27] Norman D. A. (1990). The Design of Everyday Things. New York, NY: Doubleday.

[ref28] Robertson D. J. Noyes E. Dowsett A. J. Jenkins R. Burton A. M. (2016). Face recognition by metropolitan police super-recognisers. PLoS One 11:e0150036. doi: 10.1371/journal.pone.0150036, 26918457 PMC4769018

[ref29] Russell R. Duchaine B. Nakayama K. (2009). Super-recognizers: people with extraordinary face recognition ability. Psychon. Bull. Rev. 16, 252–257. doi: 10.3758/PBR.16.2.252, 19293090 PMC3904192

[ref30] Schroff F. Kalenichenko D. Philbin J. (2015). Facenet: A unified embedding for face recognition and clustering. In Proceedings of the IEEE conference on computer vision and pattern recognition. Piscataway, NJ: IEEE. 815–823.

[ref31] Steblay N. Dysart J. Fulero S. Lindsay R. C. (2001). Eyewitness accuracy rates in sequential and simultaneous lineup presentations: a meta-analytic comparison. Law Hum. Behav. 25, 459–473. doi: 10.1023/a:1012888715007, 11688368

[ref32] Steblay N. K. Dysart J. E. Wells G. L. (2011). Seventy-two tests of the sequential lineup superiority effect: a meta-analysis and policy discussion. Psychol. Public Policy Law 17, 99–139. doi: 10.1037/a0021650

[ref33] Sweller J. (1988). Cognitive load during problem solving: effects on learning. Cogn. Sci. 12, 257–285.

[ref34] Towler A. Kemp R. I. Burton A. M. Dunn J. D. Wayne T. Moreton R. . (2019). Do professional facial image comparison training courses work? PLoS One 14:e0211037. doi: 10.1371/journal.pone.0211037, 30759105 PMC6373902

[ref35] Vicente K. J. (2003). The Human Factor: Revolutionizing the Way People Live with Technology. New York, NY: Routledge.

[ref36] Wells G. L. (1984). The psychology of lineup identifications 1. J. Appl. Soc. Psychol. 14, 89–103.

[ref370] Wells G. L. Steblay N. K. Dysart J. E. (2015). Double-blind photo lineups using actual eyewitnesses: an experimental test of a sequential versus simultaneous lineup procedure. Law Hum. Behav. 39, 1–14. doi: 10.1037/lhb0000096 24933175

[ref37] Wells G. L. Rydell S. M. Seelau E. P. (1993). The selection of distractors for eyewitness lineups. J. Appl. Psychol. 78, 835–844.

[ref38] Wells G. L. Seelau E. P. (1995). Eyewitness identification: psychological research and legal policy on lineups. Psychol. Public Policy Law 1, 765–791.

[ref39] Wells G. L. Small M. Penrod S. Malpass R. S. Fulero S. M. Brimacombe C. A. E. (1998). Eyewitness identification procedures: recommendations for lineups and photospreads. Law Hum. Behav. 22, 603–647. doi: 10.1023/A:1025750605807

[ref40] Wells G. L. Steblay N. K. Dysart J. E. (2015). Double-blind photo lineups using actual eyewitnesses: an experimental test of a sequential versus simultaneous lineup procedure. Law Hum. Behav. 39, 1–14. doi: 10.1037/lhb0000096, 24933175

[ref41] Wells G. L. Turtle J. W. (1986). Eyewitness identification: the importance of lineup models. Psychol. Bull. 99, 320–329.

[ref42] White D. Dunn J. D. Schmid A. C. Kemp R. I. (2015). Error rates in users of automatic face recognition software. PLoS One 10:e0139827. doi: 10.1371/journal.pone.0139827, 26465631 PMC4605725

[ref43] White D. Kemp R. I. Jenkins R. Burton A. M. (2014). Feedback training for facial image comparison. Psychon. Bull. Rev. 21, 100–106. doi: 10.3758/s13423-013-0475-3, 23835616

[ref44] White D. Rivolta D. Burnton M. A. Al-Janabi S. Palermo R. (2017). Face matching impairment in developmental prosopagnosia. Q. J. Exp. Psychol. 70, 287–297. doi: 10.1080/17470218.2016.1173076 27042880

[ref45] Wickens C. D. Liu Y. Gordon-Becker S. (2004). An Introduction to Human Factors Engineering. 2nd Edn. Upper Saddle River, NJ: Pearson Education.

